# Improved plant yield of potato through exogenously applied potassium fertilizer sources and biofertilizer

**DOI:** 10.1186/s13568-023-01627-7

**Published:** 2023-11-08

**Authors:** Ahmed Fathy Yousef, Ahmed Mahmoud Ali, Mohamed AbdAllah Azab, Sobhi F. Lamlom, Hassan Mohamed Al-Sayed

**Affiliations:** 1 Department of Horticulture, College of Agriculture, University of Al-Azhar (branch Assiut), Assiut, 71524 Egypt; 2 Department of Soils and Water Sciences, Faculty of Agriculture, Al-Azhar University (Assiut Branch), Assiut, 71524 Egypt; 3 Horticulture Department, Faculty of Agriculture (Assiut branch), Vegetable Sciences, Al-Azhar University, Assiut, 71524 Egypt; 4 Plant Production Department, Faculty of Agriculture Saba Basha, Alexandria University, Alexandria, 21531 Egypt

**Keywords:** Filter cake, Feldspar, Macronutrients uptake, Macronutrients available, Total tuber weight

## Abstract

Excessive usage of chemical fertilizers has detrimental effects on the environment and the safety of food. Conversely, utilizing organic fertilizers such as sage offers several advantages, including cost-effectiveness, soil enhancement, and promotion of root development. A two-year field experiment was conducted to investigate the impact of different potassium fertilizer sources and biofertilizers (specifically *Bacillus cereus* (MBc)) on potato plants. The experiment employed a split-plot design with three replicates, where the main plot factor was MBc (with and without), and the subplot factor was the sources of potassium fertilizer (control without K fertilizer, 100% Feldspar (FD), 100% Filter cake (FC), 75% FD + 25% FC, 25% FD + 75% FC, and 50% FD + 50% FC). The purpose was to examine the growth response of potato plants to these treatments. The results indicated that all treatments increased plant height, stem count, and tuber dry matter compared to the control. Furthermore, all treatments exhibited a higher uptake of macronutrients (N, P, and K) compared to the control. Notably, the plants treated with 100FC combined with MBc showed a significant 104.74% increase in total tuber weight compared to the control treatment. Additionally, the addition of 100FC with MBc significantly enhanced the availability of N, P, and K by 73.13%, 110.33%, and 51.88% respectively, compared to the control treatment. Apart from the biofertilizers, the individual application of FC and its combination with FD also demonstrated positive effects on soil fertility, potato growth, and yield.

## Introduction

The potato (*Solanum tuberosum* L.) known for its global significance as the third most consumed food crop, has found a special place in the fields of Egypt, becoming one of its most important solanaceous vegetable crops (Kaguongo et al. 2013). Packed with essential nutrients—carbohydrates, proteins, vitamins, and minerals—potatoes are not just a staple but a lifeline for millions, both locally and across the world (Singh and Kaur 2016). Egypt’s Ministry of Agriculture and Land Reclamation reported a remarkable expansion in potato cultivation from 149 thousand hectares in 2010 to 183 thousand hectares in 2015, yielding a bountiful 5 million tons (Ali et al. 2021a). However, with a burgeoning global population, the pressure to increase potato production is mounting. The key to meeting this challenge lies in strategic fertilization practices, as underscored by recent research (Vilvert et al. 2022).

Amid this backdrop, a green revolution is taking root. Interest is surging in the use of natural, organic, and bio-fertilizers to boost soil fertility and amplify crop productivity (Kumar et al. 2022). In Egypt, innovative farmers are turning to alternatives that promise not only to reduce the excessive use of chemical fertilizers but also to mitigate the environmental toll exacted by modern agriculture. These alternatives include natural resources like feldspar, organic wonders like filter cake, and the intriguing realm of bio-fertilizers (Ali et al. 2021a; Tantawy et al. 2022).

Traditionally, farmers have saturated their fields with chemical fertilizers, particularly potassium, to maximize yields. This practice, while effective in the short term, has led to nutrient scarcity, soaring costs, and environmental degradation (Dwivedi et al. 2017). Feldspar, with its resistance to weathering, offers a beacon of hope. Paired with organic ameliorants and bio-fertilizers, it emerges as an economical and eco-friendly approach to soil enrichment (Shabrawy and Ragab 2019). As feldspar weathers on the Earth’s surface, it transforms, releasing vital nutrients for plants and fostering the formation of secondary clay minerals (Parker 1995). These weathered feldspars are treasure troves of nutrient metals, accessible to plants as they weather in the soil (Mishra and Samant 2021). In a virtuous cycle, clay minerals derived from weathered feldspars fortify agriculture by enhancing soil water retention and nutrient availability (Kumari and Mohan 2021).

On another front, filter cake (FC), a byproduct of sugar production, emerges as a potent organic ally. Its unassuming appearance belies its richness—sugar, fiber, coagulated colloids, inorganic salts, and tiny particles of dirt (Sasy and Abu-Ellail 2021). But the treasure trove doesn’t end there. FC boasts plant growth regulators, auxins, enzymes, vitamins, and hormones, all of which breathe life into agricultural soils, enhancing texture, structure, organic content, water-holding capacity, and aeration (El-Tayeh et al. 2019). In essence, filter cake is the embodiment of an organic waste product becoming a life-giving force for soil and crops (Goncalves et al. 2018).

And then there are bio-fertilizers, the unsung heroes of sustainable agriculture. Bacillus, a genus of beneficial bacteria, steps into the limelight, forming associations with plant roots and rhizospheres, and weaving biofilms that nurture plant growth (Beauregard et al. 2013; El-Sawah et al. 2021; Gao et al. 2020). The application of Bacillus-based fertilizers goes beyond the superficial; it unlocks nutrients in the rhizosphere (Yousef et al. 2020; Youssef et al. 2021), keeps pathogenic microbes at bay, and activates the plant’s innate defenses against pests (García-Fraile et al. 2015; Kang et al. 2015).

Research consistently underscores the effectiveness of organic and natural sources in yielding high crop yields, improving economic outcomes, and fortifying soil fertility over the long term (Kihara et al. 2022). The clarion call is clear: embracing environmentally friendly alternatives is not just a choice; it’s imperative. It’s a path that not only reduces environmental pollution but also paves the way for the flourishing of plants, bridging the chasm between human sustenance and ecological stewardship. As the story unfolds, it is a testament to the power of science to transform the very roots of our sustenance, offering a brighter and more sustainable future for agriculture.

The hypothesis of the current study was the application of organic fertilizers, biofertilizers, and their combination holds the potential to enhance various aspects of potato cultivation, specifically targeting plant growth, nutrient uptake, tuber yield, and overall quality. This improvement in potato plants is achieved by harnessing potassium fertilizer sources. Our research seeks to address this hypothesis through a set of well-defined objectives, which can be summarized as follows: The first objective of this study is to comprehensively examine alterations in soil properties and nutrient availability stemming from the individual or combined application of filter cake (FC) and feldspar (FD). By assessing their impact on soil dynamics, we aim to gain insights into how these amendments influence potato growth and ultimately yield. The second objective is to explore the feasibility of substituting chemical fertilizers with natural or organic alternatives, in conjunction with biofertilization practices. This objective involves a dual approach-evaluating the potential for partial replacement as well as complete substitution of chemical fertilizers. By doing so, we aim to establish environmentally sustainable and resource-efficient potato cultivation practices.

## Materials and methods

### Experimental site and weather conditions

**Fig. 1:**
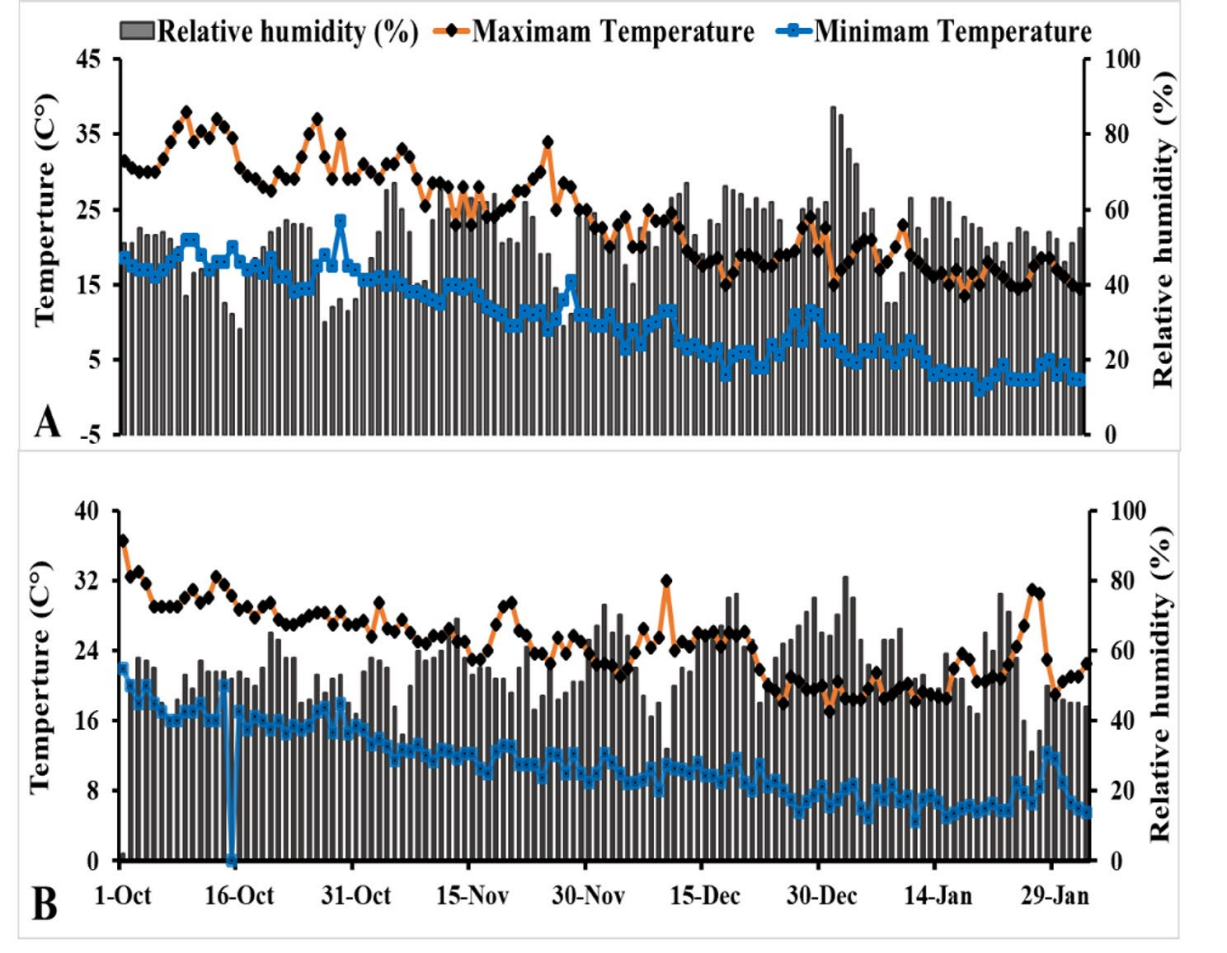
Weather conditions [Maximum temperature, Minimum temperature, and Relative humidity] during the growing seasons [2021/2022 (**A**) and 2022/2023 (**B**)] of Potato cultivation

According to the Köppen-Geiger climate map, the climate in the experimental region, particularly El-Usayrat city, Sohag governorate in Egypt (located at 26°22′N 31°50′E), is classified as extremely hot and dry during the summer months, while winters are characterized by cold temperatures (Beck et al. 2018). Figure 1 displays the maximum and minimum temperatures, as well as the relative humidity recorded during the growing seasons of 2021/2022 and 2022/2023. The atmospheric data utilized in this study were gathered from Weather-Online (2023).

Figure 1. Weather conditions [Maximum temperature, Minimum temperature, and Relative humidity] during the growing seasons [2021/2022 (A) and 2022/2023 (B)] of Potato cultivation.

### Experimental design and treatments

The field experiments were conducted during the winter seasons of 2021/2022 and 2022/2023. The experimental plots had dimensions of 3 m in width and 3.5 m in length. Certified potato tubers of the Rosetta variety were obtained from the National Research Center, Giza, Egypt, and were stored at room temperature (25 °C) for 15 days until a uniform seedling stage was achieved (cultured tubers). The tubers were then manually planted at a depth of 10–15 cm with a spacing of 30 cm between hills.

Potassium solubilizing bacteria [*Bacillus cereus* (ATCC® 14,579™)] were obtained from the National Research Center, Giza, Egypt, in the form of a liquid broth culture containing 5 × 10^9^ CFU/mL, which was purchased from (https://www.atcc.org/products/14579). These bacteria were added to the potato plants immediately after emergence before irrigation and repeated one week later to ensure their effectiveness. The soil was sterilized before planting using 238 kg ha^− 1^ of agricultural sulfur produced by Abu Qir Fertilizer and Chemical Industries.

The experimental design was a split-plot design with three replicates. The main plot was allocated by the soil application of bio-fertilizer (with and without), while subplot occupied by application sources of potassium at different rates treatments as follows: Without adding any K fertilizer (control, CK), 100% Feldspar (100FD), 100% Filter cake (100FC), 75% Feldspar + 25% Filter cake (75FD + 25FC). 25% Feldspar + 75% Filter cake (25FD + 75FC), 50% Feldspar + 50%Filter cake (50FD + 50FC). According to the Egyptian Ministry of Agriculture and Land Reclamation, a hectare of potato plants requires 171.5 kg of potassium units. For each plot, a total of 171.5 K units from either filter cake (FC) or feldspar (FD) were individually added to the soil before planting, either separately or in various combinations as shown in Table (1). Some chemical analysis of the potassium sources (Feldspar (FD) and Filter Cake (FC)) is presented in Table (2).

**Table 1:** Treatment labels of the potassium alternative sources additions to the studied soil

Factors		Treatments	Labels
Main plot	(*Bacillus cereus*)	With	MBc+
		Without	MBc-
Subplot	Potassium alternative sources	Without adding any K fertilizer (control)	Control
		100% Feldspar (FD)	100FD
		100% Filter cake (FC)	100FC
		75% Feldspar (FD) + 25% Filter cake (FC)	75FD + 25FC
		25% Feldspar (FD) + 75% Filter cake (FC)	25FD + 75FC
		50% Feldspar (FD) + 50%Filter cake (FC)	50FD + 50FC

**Table 2:** Some chemical properties of the potassium sources

Potassium sources	Chemical properties			Macro nutrients (g kg^− 1^)					
				Total			Available		
	pH (Susp. 1:2.5 soil-water)	EC_e_ (1:1 dSm^− 1^)	Organic matter (g kg^− 1^)	N	P	K	N	P	K
Filter Cake (FC)	7.55	9.76	645.60	19.10	15.90	20.30	3.36	2.80	3.62
Feldspar (FD)	9.04	8.02				50.00			1.44

### Soil and plant analysis

Various physicochemical soil properties of the field experiment were analyzed following the methods described by Carter and Gregorich (2007) and are listed in Table 3. In each season, surface soil samples (0 − 30 cm) were randomly collected. The samples were air-dried, crushed, and sieved through 2 mm mesh sieve. Soil texture was determined by the pipette as described by Page et al. (1982).

The pH of the soil was determined by estimating the 1:2.5 soil-to-water suspension using a Beckman pH meter and electrical conductivity (EC) at a ratio of 1:2.5 was measured using a salt bridge (Jackson 1973). The total calcium carbonate content (CaCO_3_) was determined using a Collins calcimeter following the method described by (Loeppert and Suarez 1996).

After the soil samples were brought to the laboratory, they were subjected to a series of preparation steps. The samples were first oven-dried at 40 °C, then crushed to pass through a 2 mm sieve, and finally ground to a particle size of less than 60 μm, following the procedures described by Madejón et al. (2006). To determine the available nitrogen (N), the soil samples were extracted with 1% K_2_SO_4_ at a ratio of 1:10. Five milliliters of the extract were distilled with the addition of 0.1 g of a mixture of magnesium oxide (MgO) and Devarda’s alloy using a micro Kjeldahl’s distilling unit (Jackson 1973). The distillate was collected in an Erlenmeyer flask containing 15 mL of boric acid (H_3_BO_3_) mixed with an indicator solution. Approximately 50 mL of distillate was collected in each flask. The available nitrogen content (NH_4_^+^ and NO_3_^−^) in the distillate was determined by titration with standardized 0.01 N sulfuric acid, following the method described by Jackson (1973). For the determination of available phosphorus (P), the soil samples were extracted with 0.5 M (NaHCO_3_) at pH 8.5 at a ratio of 1:10. The extracted P was measured using a spectrophotometer (JENWAY 6305 UV/Visible spectrophotometer, U.K.) with the stannous chloride phosphomolybdic-sulfuric acid system, as described by Jackson (1973). Available potassium (K) was extracted using 1 M ammonium acetate at pH 7 at a ratio of 1:10. The extracted K was measured by flame photometry using a BWB model BWB-XP, 5-channel, JENWAY, model: PFP7, U.K., according to the method outlined by Jackson (1973). To measure nutrient concentrations in potato tuber, a mixture of 7:3 ratio of sulfuric to perchloric acids was used to digest the dried ground plant material. Total N, P and K were determined as described by Jackson (1973).

**Table 3:** Some physical and chemical characteristics of soil experimental

Parameters			Values	
			2021/2022	2022/2023
Particle size distribution	Sand (g kg.^−1^)		535	542
	Silt (g kg.^−1^)		223	254
	Clay (g kg.^−1^)		242	204
	Texture grade		Sandy clay loam	Sandy clay loam
Chemical properties	pH (Susp. 1:2.5 soil-water)		8.04	8
	EC_e_ (1:1 dSm^− 1^)		1.40	1.48
	Organic matter (g kg^− 1^)		13.80	15.11
	CaCO_3_ (g kg^− 1^)		14	15
Macro nutrients	Total (mg kg^− 1^)	N	300	310
		P	297	300
		K	394	400
	Available (mg kg^− 1^)	N	53.00	61.00
		P	8.50	8.93
		K	92.60	101.00

The values presented in the table represent the average of four replicates for each measurement

### Data collection

During the harvest stage, plant samples were collected on 28th January 2022, and 2nd February 2023. The growth parameters, including plant height and the number of branches, were recorded. The tuber samples were cleaned and washed with both tap water and distilled water, followed by air drying. Subsequently, they were dried in an oven at a temperature of 70 °C until reaching a constant weight. Afterward, the dried tuber samples were ground and stored for chemical analysis. Potato tuber samples were obtained at the end of each season, specifically selecting tubers from a full line with a length of 3.5 m, taken from the middle plots. These tubers were collected to estimate the fresh potato yield. In the laboratory, tuber samples were taken randomly and sorted based on their size, which included categories such as large (> 6.5 cm), medium (5.0 to 6.5 cm), and small (2.5 to 5.0 cm).

After harvesting, each tuber grade was weighed, and the number of tubers in each grade was counted. The tubers were then cleaned, washed with tap and distilled water, and air dried. Subsequently, they were dried in an oven at 70 °C until a constant weight was achieved. The dry yield was recorded, and the tubers were ground and stored for further chemical analysis.

### Statistical **analysis**

The data obtained from these experiments were subjected to statistical analysis using the software Statistix 8.1. Two-way ANOVA (Analysis of Variance) was performed on the growth parameters and yield traits to examine the significance of the effects of different factors. Duncan’s multiple range tests were employed to further explore and compare the means that showed significant differences. These tests allow for a detailed examination of the variations between treatment means with a 95% confidence level (Gomez and Gomez 1984).

## Results

### Morphological **traits**

The results of the study indicate that the choice of potassium sources and the application of biofertilizer had a significant impact on the morphology of potato plants (Table 4). During the first and second growing seasons, the combination of 25% filter cake and 75% fertilizer (25FD + 75FC) resulted in the tallest plants (75.00 and 75.54 cm), with no significant differences compared to other treatments except the control (64.78 and 65.32 cm), respectively. The application of biofertilizer led to the highest plant height (75.69 and 76.23 cm) during the first and second growing seasons, respectively. Considering different potassium sources and biofertilizer cumulatively, the plants treated with 75% filter cake and 25% fertilizer along with the biofertilizer exhibited the maximum plant height (80.22 and 80.76 cm) during the first and second growing seasons, respectively.

In terms of the number of stems, plants treated with 100% fertilizer (100FD) had the highest number (3.89) during the first growing season. The biofertilizer significantly influenced this trait, resulting in the highest average number of stems (3.52 and 3.75) in the plants treated with the biofertilizer during the first and second growing seasons, respectively. The interaction between potassium sources and biofertilizer did not have a notable impact on the number of stems in both growing seasons.

Plants treated with 100% filter cake (100% FC) had the highest dry matter of tuber (14.14 and 16.88 tons ha^− 1^) in both growing seasons, respectively. The biofertilizer had a significant impact on the dry matter of tuber only in the first growing season, but not in the second growing season. The highest dry matter of tuber was observed in the plants treated with biofertilizer (12.57 and 13.56tons ha^− 1^) during the first and second seasons, respectively. The interactive effect of potassium sources and biofertilizer resulted in substantial variations in the dry matter of tuber, with the highest dry matter recorded in the plants treated with 75% filter cake and 25% fertilizer along with the biofertilizer (19.51 and 20.50 tons ha^− 1^) during the first and second growing seasons, respectively.

**Table 4:** The integrated effect of feldspar (FD), Filter cake (FC) treatments and their combinations with biofertilizer on growth variables of potato plants after two successive seasons (2021/2022–2022/2023)

Treatments		Plant height (cm)			No of stem			Dry matter of tuber (ton ha^− 1^)		
		MBc(-)	MBc(+)	Mean (OFs)	MBc(-)	MBc(+)	Mean (OFs)	MBc(-)	MBc(+)	Mean (OFs)
**2021/2022**	**Control**	54.78c	74.78ab	64.78b	2.33a	3.33a	2.83a	4.60d	6.17 cd	5.38c
	**100FD**	61.78bc	76.00ab	68.89ab	3.78a	3.89a	3.84a	11.14bcd	8.82bcd	9.98b
	**100FC**	68.11abc	76.11ab	72.11ab	3.00a	3.33a	3.17a	15.33ab	12.95ab	14.14a
	**75FD + 25FC**	60.33bc	80.22a	70.28ab	2.67a	3.33a	3.00a	8.66bcd	19.51a	14.09a
	**25FD + 75FC**	74.78ab	75.22ab	75.00a	2.78a	3.45a	3.11a	11.99bc	15.11ab	13.55ab
	**50FD + 50FC**	66.78abc	71.78ab	69.28ab	3.00a	3.78a	3.39a	10.47bcd	12.87ab	11.67ab
**Mean (MBc)**		64.43b	75.69a		2.93b	3.52a		10.36b	12.57a	
LSD (*P ≤ 0.05*)		A = 3.89	B = 10.12	AB = 16.71	A = 0.53	B = 1.38	AB = 2.27	A = 1.56	B = 4.06	AB = 6.69
**2022/2023**	**Control**	55.32c	75.32ab	65.32b	2.56a	3.56a	3.06a	5.58c	7.16bc	6.37c
	**100FD**	62.32bc	76.54ab	69.43ab	4.01a	4.12a	4.07a	12.13abc	9.80bc	10.97bc
	**100FC**	68.65abc	76.65ab	72.65ab	3.23a	3.56a	3.40a	19.82a	13.94abc	16.88a
	**75FD + 25FC**	60.87bc	80.76a	70.82ab	2.90a	3.56a	3.23a	9.65bc	20.50a	15.08ab
	**25FD + 75FC**	75.32ab	75.76ab	75.54a	3.01a	3.68a	3.34a	12.98abc	16.10ab	14.54ab
	**50FD + 50FC**	67.32abc	72.32ab	69.82ab	3.23a	4.01a	3.62a	11.46abc	13.86abc	12.66ab
**Mean (MBc)**		64.97b	76.23a		3.16b	3.75a		11.94a	13.56a	
LSD (*P ≤ 0.05*)		A = 3.89	B = 10.12	AB = 16.71	A = 0.53	B = 1.38	AB = 2.27	A = 2.12	B = 5.52	AB = 9.12

The values shown in table are means three replicates. Means followed by the same letters are non-significantly different (*p ≤ 0.05*). Where MBc(-) = Without biofertilizer (*Bacillus cereus*); MBc(+) = With biofertilizer (*Bacillus cereus*); Control = Non-fertilizer; 100FD = 100% Feldspar; 100FC = 100% Filer Cake; 75FD + 25FC = 75% Feldspar + 25% Filer Cake; 25FD + 75FC = 25% Feldspar + 75% Filer Cake; 50FD + 50FC = 50% Feldspar + 50% Filer Cake

### Macro nutrients Uptake

The results indicate that the choice of potassium sources and bio-fertilizer had a significant impact on the uptake of macro nutrients by potato tubers, as shown in Table 5. During the first and second growing seasons, potato plants treated with 100% FC exhibited the highest nitrogen uptake (427.43 and 428.29 kg ha^− 1^), comparable to the treatment 25% FD + 75% FC (326.67 and 327.53 kg ha^− 1^). The bio-fertilizer treatment MBc + also led to high nitrogen uptake (291.53 and 291.39 kg ha^− 1^), without significant differences compared to MBc. Cumulatively, the highest nitrogen uptake occurred in plants treated with 100% FC without the bio-fertilizer (518.86 and 519.72 kg ha^− 1^) during the first and second growing seasons, respectively.

Phosphorus uptake was highest in plants treated with 100% FC during both growing seasons (107.38 and 108.30 kg ha^− 1^). The bio-fertilizer significantly increased phosphorus uptake, with the highest values observed in bio-fertilizer-treated plants (81.86 and 82.79 kg ha^− 1^). The combination of potassium sources and bio-fertilizer had substantial effects on phosphorus uptake, with the highest uptake occurring when using 75% filter cake and 25% feldspar along with the bio-fertilizer (130.11 and 131.04 kg ha^− 1^) during the first and second growing seasons, respectively.

Potassium uptake was highest in plants treated with 75% feldspar and 25% filter cake in both growing seasons (1037.60 and 1038.30 kg ha^− 1^). The bio-fertilizer also significantly impacted potassium uptake, with the highest uptake observed in plants treated with MBc+ (860.38 and 861.05 kg ha^− 1^). The interactive effect of potassium sources and bio-fertilizer resulted in substantial variations in potassium uptake, with the highest uptake occurring when using 75% FC and 25% FD along with the bio-fertilizer (1563.80 and 1564.50 kg ha^− 1^) during the first and second growing seasons, respectively.

**Table 5:** The integrated effect of feldspar (FD), Filter cake (FC) treatments and their combinations with biofertilizer on the uptake (kg ha^− 1^) of nitrogen (N), phosphorous (P), and potassium (K) of potato tubers after two successive seasons (2021/2022–2022/2023)

Treatments		Uptake (N) kg ha^− 1^			Uptake (P) kg ha^− 1^			Uptake (K) kg ha^− 1^		
		MBc(-)	MBc(+)	Mean (OFs)	MBc(-)	MBc(+)	Mean (OFs)	MBc(-)	MBc(+)	Mean (OFs)
**2021/2022**	**Control**	80.20e	105.06de	92.63c	14.43e	27.31de	20.87c	201.8e	299.4de	250.6c
	**100FD**	273.10b-e	210.14b-e	241.62b	45.63cde	52.65cde	49.14bc	625.7b-e	527.4cde	576.6bc
	**100FC**	518.86a	336.00abc	427.43a	125.51ab	89.24a-d	107.38a	1192.1ab	859.5bcd	1025.8a
	**75FD + 25FC**	159.68cde	415.71ab	287.70b	55.68b-e	130.11a	92.89a	511.4cde	1563.8a	1037.6a
	**25FD + 75FC**	286.62b-e	366.71abc	326.67ab	76.69a-e	100.51abc	88.60ab	720.7b-e	1064.7abc	892.7ab
	**50FD + 50FC**	248.29b-e	309.59a-d	278.94b	69.32a-e	91.33a-d	80.33ab	658.6b-e	847.4bcd	753.0ab
**Mean (MBc)**		261.13a	290.53a		64.54b	81.86a		651.70b	860.38a	
LSD (*P ≤ 0.05*)		A = 53.24	B = 138.43	AB = 228.52	A = 16.75	B = 43.57	AB = 71.92	A = 143.42	B = 372.94	AB = 615.64
**2022/2023**	**Control**	81.06e	105.92de	93.49c	15.36e	28.24de	21.80c	202.4e	300.1de	251.3c
	**100FD**	273.96b-e	211.00b-e	242.48b	46.56cde	53.58cde	50.07bc	626.4b-e	528.1cde	577.2bc
	**100FC**	519.72a	336.86abc	428.29a	126.43ab	90.17a-d	108.30a	1192.7ab	860.2bcd	1026.5a
	**75FD + 25FC**	160.54cde	416.57ab	288.56b	56.60b-e	131.04a	93.82a	512.0cde	1564.5a	1038.3a
	**25FD + 75FC**	287.48b-e	367.57abc	327.53ab	77.61a-e	101.44abc	89.53ab	721.4b-e	1065.4abc	893.4ab
	**50FD + 50FC**	249.15b-e	310.45a-d	279.80b	70.25a-e	92.26a-d	81.25ab	659.3b-e	848.1bcd	753.7ab
**Mean (MBc)**		261.99a	291.39a		65.47b	82.79a		652.37b	861.05a	
LSD (*P ≤ 0.05*)		A = 53.24	B = 138.43	AB = 228.52	A = 16.75	B = 43.57	AB = 71.92	A = 143.42	B = 372.94	AB = 615.64

The values shown in table are means three replicates. Means followed by the same letters are non-significantly different (*p ≤ 0.05*). Where MBc(-) = Without biofertilizer (*Bacillus cereus*); MBc(+) = With biofertilizer (*Bacillus cereus*); Control = Non-fertilizer; 100FD = 100% Feldspar; 100FC = 100% Filer Cake; 75FD + 25FC = 75% Feldspar + 25% Filer Cake; 25FD + 75FC = 25% Feldspar + 75% Filer Cake; 50FD + 50FC = 50% Feldspar + 50% Filer Cake

### Yield traits

The results indicate that the choice of potassium sources and bio-fertilizer had a significant impact on the yield traits of potato plants, as shown in Table 6. During the first and second growing seasons, plants treated with 100FC exhibited the highest weight of large tubers (26.04 and 26.46 tons ha^− 1^), respectively. Regarding the bio-fertilizer, it resulted in the highest weight of large tubers (21.76 and 22.19 tons ha^− 1^) during the first and second growing seasons, respectively. Cumulatively, when considering different potassium sources and bio-fertilizer, plants treated with 100FC along with the bio-fertilizer exhibited the maximum weight of large tubers (28.00 and 28.43 tons ha^− 1^) during the first and second growing seasons, respectively.

For the weight of medium tubers, plants treated with 100FD showed the highest weight (11.52 and 11.75 tons ha^− 1^) with potassium sources during the first and second growing seasons, respectively. The bio-fertilizer significantly influenced this trait, resulting in the highest weight of medium tubers (9.42 and 9.65 tons ha^− 1^) in plants treated with the bio-fertilizer during the first and second growing seasons, respectively, without significant differences compared to MBc-. The interaction between potassium sources and bio-fertilizer led to substantial variations in the weight of medium tubers, with the highest weight observed in plants treated with 100FC without bio-fertilizer (11.69 and 11.92 tons ha^− 1^) during the first and second growing seasons, respectively.

Based on the results in Table 6, the highest weight of small tubers (6.20 and 6.61 tons ha^− 1^) was recorded in plants treated with 50% feldspar and 50% filter cake in both growing seasons, respectively, with no significant differences among the other treatments. The bio-fertilizer also significantly impacted the weight of small tubers in both growing seasons, with the highest weight (6.19 and 6.60 tons ha^− 1^) observed in plants treated with MBc + in both growing seasons, respectively. The interactive effect of potassium sources and bio-fertilizer resulted in substantial variations in small tuber weight, with the highest weight observed in plants treated with 50FC with 50FD along with the bio-fertilizer (6.83 and 7.24 tons ha^− 1^) during the first and second growing seasons, respectively, with no significant differences among the other treatments.

**Table 6:** The integrated effect of feldspar (FD), Filter cake (FC) treatments and their combinations with biofertilizer on the graded weight (ton ha^− 1^) of potato tubers after two successive seasons (2021/2022–2022/2023)

Treatments		Large Tuber weight (ton ha^− 1^)			Medium Tuber weight (ton ha^− 1^)			Small Tuber weight (ton ha^− 1^)		
		MBc(-)	MBc(+)	Mean (OFs)	MBc(-)	MBc(+)	Mean (OFs)	MBc(-)	MBc(+)	Mean (OFs)
**2021/2022**	**Control**	7.99f	10.25f	9.12e	4.95e	6.11de	5.53d	3.82a	5.38a	4.60a
	**100FD**	16.93e	19.72d	18.32d	8.72bc	10.42abc	9.57bc	3.85a	6.42a	5.13a
	**100FC**	24.07bc	28.00a	26.04a	11.69a	11.35a	11.52a	6.09a	6.13a	6.11a
	**75FD + 25FC**	18.82de	22.76c	20.79c	11.26a	9.27abc	10.27ab	5.16a	6.18a	5.67a
	**25FD + 75FC**	19.40de	26.25ab	22.83b	9.31abc	11.18ab	10.25ab	5.17a	6.22a	5.69a
	**50FD + 50FC**	19.01de	23.59c	21.30bc	8.20 cd	8.20 cd	8.20c	5.56a	6.83a	6.20a
**Mean (MBc)**		17.70b	21.76a		9.02a	9.42a		4.94b	6.19a	
LSD (*P ≤ 0.05*)		A = 0.61	B = 1.58	AB = 2.601	A = 0.59	B = 1.53	AB = 2.52	A = 0.76	B = 1.98	AB = 3.26
**2022/2023**	**Control**	8.42f	10.68f	9.55e	5.18e	6.34de	5.76d	4.23a	5.79a	5.01a
	**100FD**	17.357e	20.15d	18.75d	8.95bc	10.65abc	9.80bc	4.26a	6.83a	5.54a
	**100FC**	24.50bc	28.43a	26.46a	11.92a	11.58a	11.75a	6.50a	6.54a	6.52a
	**75FD + 25FC**	19.25de	23.19c	21.22c	11.49a	9.50abc	10.50ab	5.57a	6.59a	6.08a
	**25FD + 75FC**	19.83de	26.68ab	23.26b	9.54abc	11.41ab	10.48ab	5.58a	6.63a	6.10a
	**50FD + 50FC**	19.44de	24.02c	21.73bc	8.43 cd	8.43 cd	8.43c	5.97a	7.24a	6.61a
**Mean (MBc)**		18.13b	22.19a		9.25a	9.65a		5.35b	6.60a	
LSD (*P ≤ 0.05*)		A = 0.61	B = 1.58	AB = 2.61	A = 0.59	B = 1.53	AB = 2.52	A = 0.76	B = 1.98	AB = 3.26

The values shown in table are means three replicates. Means followed by the same letters are non-significantly different (*p ≤ 0.05*). Where MBc(-) = Without biofertilizer (*Bacillus cereus*); MBc(+) = With biofertilizer (*Bacillus cereus*); Control = Non-fertilizer; 100FD = 100% Feldspar; 100FC = 100% Filer Cake; 75FD + 25FC = 75% Feldspar + 25% Filer Cake; 25FD + 75FC = 25% Feldspar + 75% Filer Cake; 50FD + 50FC = 50% Feldspar + 50% Filer Cake

**Fig. 2:**
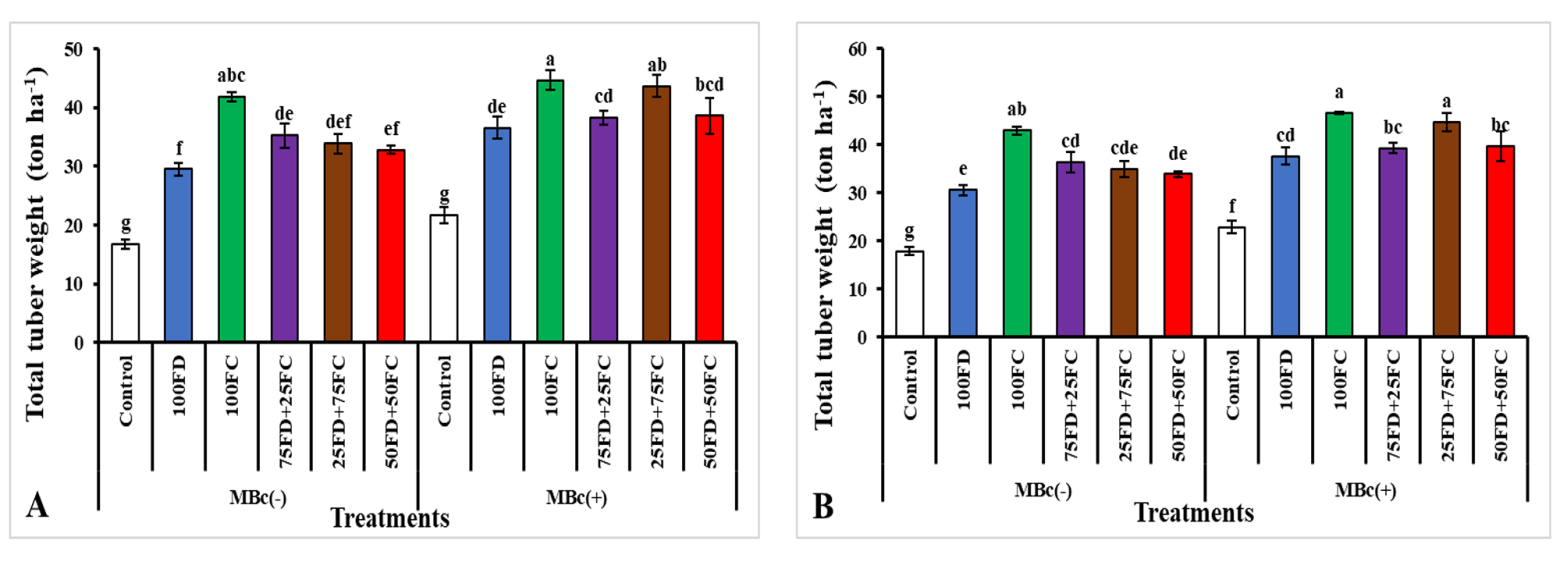
The integrated effect of feldspar (FD), Filter cake (FC) treatments and their combinations with biofertilizer on total tuber weight (ton ha^− 1^) of potato in season 2021/2022 (**A**), total tuber weight (ton ha^− 1^) of potato in season 2022/2023 (**B**). Where MBc(-) = Without biofertilizer (*Bacillus cereus*); MBc(+) = With biofertilizer (*Bacillus cereus*); Control = Non-fertilizer; 100FD = 100% Feldspar; 100FC = 100% Filer Cake; 75FD + 25FC = 75% Feldspar + 25% Filer Cake; 25FD + 75FC = 25% Feldspar + 75% Filer Cake; 50FD + 50FC = 50% Feldspar + 50% Filer Cake. The values shown in figure are means three replicates. Means followed by the same letters are non-significantly different (*p ≤ 0.05)*

The results presented in Fig. 2 indicate that the choice of potassium sources and bio-fertilizer had a significant impact on the yield traits of potato plants. Considering the cumulative effect of different potassium sources and bio-fertilizer, the plants treated with 100FC with the bio-fertilizer exhibited the maximum total tuber weight (44.68 and 46.54 ton ha^− 1^) during the first and second growing seasons (Fig. 2A and B), respectively. However, there were no significant differences observed between the treatments of 100FC alone and 25FD + 75FC with MBc in both seasons. On the other hand, the plants under the control treatment without the bio-fertilizer (MBc) recorded the minimum total tuber weight (16.77 and 17.84 ton ha^− 1^) during the first and second growing seasons, respectively.

Figure 2. The integrated effect of feldspar (FD), Filter cake (FC) treatments and their combinations with biofertilizer on total tuber weight (ton ha^− 1^) of potato in season 2021/2022 (A), total tuber weight (ton ha^− 1^) of potato in season 2022/2023 (B). Where MBc(-) = Without biofertilizer (*Bacillus cereus*); MBc(+) = With biofertilizer (*Bacillus cereus*); Control = Non-fertilizer; 100FD = 100% Feldspar; 100FC = 100% Filer Cake; 75FD + 25FC = 75% Feldspar + 25% Filer Cake; 25FD + 75FC = 25% Feldspar + 75% Filer Cake; 50FD + 50FC = 50% Feldspar + 50% Filer Cake. The values shown in table are means three replicates. Means followed by the same letters are non-significantly different (*p ≤ 0.05*).

### Post-harvest fertility status of soil

**Fig. 3:**
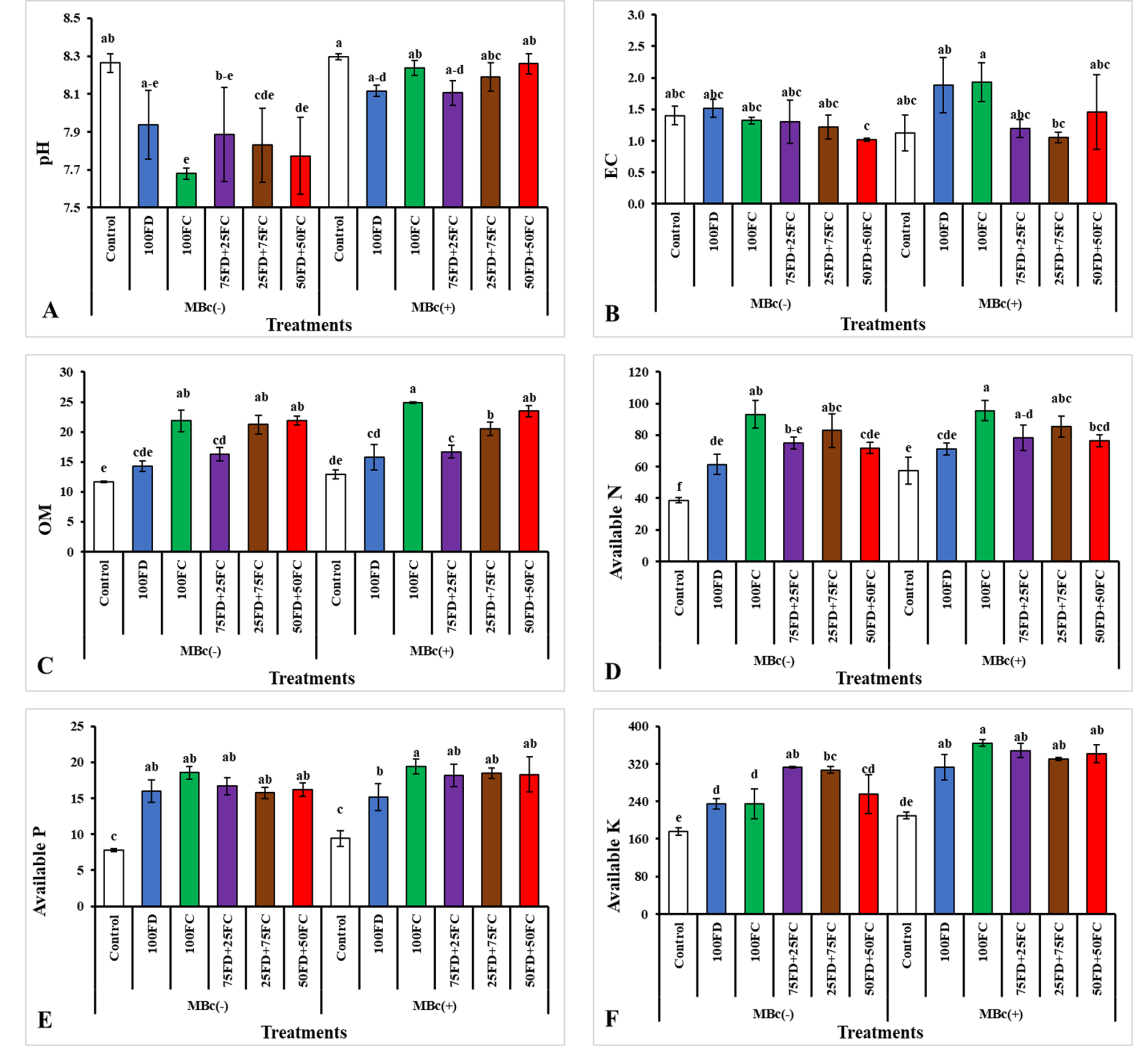
The integrated effect of feldspar (FD), Filter cake (FC) treatments and their combinations with biofertilizer on soil reaction pH (**A**), Electrical conductivity (**B**), organic matter (OM) (**C**), available nitrogen (**D**), available phosphorus (**E**), and available potassium (**F**) (mg kg^− 1^) after successive season 2021/2022. Where MBc(-) = Without biofertilizer (*Bacillus cereus*); MBc(+) = With biofertilizer (*Bacillus cereus*); Control = Non-fertilizer; 100FD = 100% Feldspar; 100FC = 100% Filer Cake; 75FD + 25FC = 75% Feldspar + 25% Filer Cake; 25FD + 75FC = 25% Feldspar + 75% Filer Cake; 50FD + 50FC = 50% Feldspar + 50% Filer Cake. The values shown in figure are means three replicates. Means followed by the same letters are non-significantly different (*p ≤ 0.05*)

**Fig. 4:**
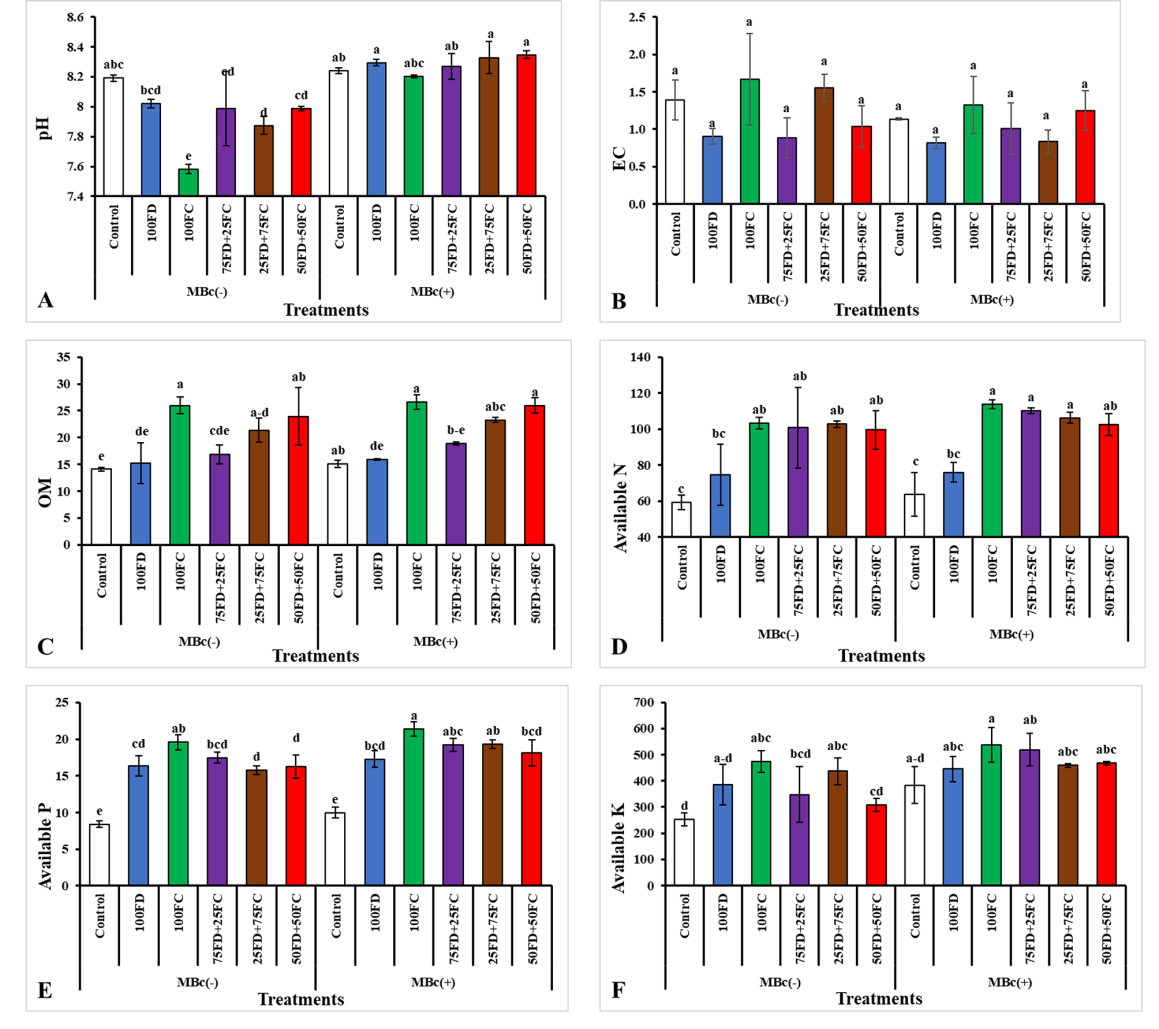
The integrated effect of feldspar (FD), Filter cake (FC) treatments and their combinations with biofertilizer on soil reaction pH (**A**), Electrical conductivity EC (**B**), organic matter (OM) (**C**), available nitrogen (**D**), available phosphorus (**E**), and available potassium (**F**) (mg kg^− 1^) after successive season 2022/2023. Where MBc(-) = Without biofertilizer (*Bacillus cereus*); MBc(+) = With biofertilizer (*Bacillus cereus*); Control = Non-fertilizer; 100FD = 100% Feldspar; 100FC = 100% Filer Cake; 75FD + 25FC = 75% Feldspar + 25% Filer Cake; 25FD + 75FC = 25% Feldspar + 75% Filer Cake; 50FD + 50FC = 50% Feldspar + 50% Filer Cake. The values shown in figure are means three replicates. Means followed by the same letters are non-significantly different (*p ≤ 0.05*)

The results presented in Figs. 3 and 4 demonstrate that the application of different potassium sources and bio-fertilizer had a significant impact on the chemical properties of the soil. Regarding soil pH, the use of 100FC resulted in a reduction in soil pH compared to the control treatment in both growing seasons. The bio-fertilizer did not play a significant role in reducing pH during either growing season (Fig. 3A, and 4 A).

The electrical conductivity (EC) of the soil was influenced by the different treatments. The maximum EC was recorded when 100FC was applied along with the bio-fertilizer (MBc) in the first season (Fig. 3B), while the second season, the highest EC was observed when 100FC was applied alone (Fig. 4B).

Figure 3. The integrated effect of feldspar (FD), Filter cake (FC) treatments and their combinations with biofertilizer on soil reaction pH (A), Electrical conductivity (B), organic matter (OM) (C), available nitrogen (D), available phosphorus (E), and available potassium (F) (mg kg^− 1^) after successive season 2021/2022. Where MBc(-) = Without biofertilizer (*Bacillus cereus*); MBc(+) = With biofertilizer (*Bacillus cereus*); Control = Non-fertilizer; 100FD = 100% Feldspar; 100FC = 100% Filer Cake; 75FD + 25FC = 75% Feldspar + 25% Filer Cake; 25FD + 75FC = 25% Feldspar + 75% Filer Cake; 50FD + 50FC = 50% Feldspar + 50% Filer Cake. The values shown in figure are means three replicates. Means followed by the same letters are non-significantly different (*p ≤ 0.05*).

The application of 100FC, with or without the bio-fertilizer, increased organic matter (OM) content. The highest OM content (24.88 and 26.64 g kg^− 1^) was recorded in the plants treated with 100FC along with MBc during the 1st and 2nd growing seasons, respectively (Figs. 3C and 4C).

In terms of nutrient availability, the application of potassium sources and bio-fertilizer, either individually or in combination, increased the levels of available nitrogen (N), phosphorus (P), and potassium (K) in the soil (Figs. 3D-F and 4D-F). Considering the interaction effect, the highest available N (95.43 and 113.86 mg kg^− 1^) was recorded when 100FC was applied along with the bio-fertilizer during the 1st and 2nd growing seasons (Fig. 3D, and 4D), respectively. For available P, the maximum values (19.42 and 21.36 mg kg^− 1^) were observed when 100FC was applied along with MBc during the 1st and 2nd growing seasons (Fig. 3E, and 4E), respectively. Similarly, the highest available K (364.00 and 536.67 mg kg^− 1^) was recorded when 100FC was applied along with MBc during the 1st and 2nd growing seasons (Fig. 3F, and 4 F), respectively.

Figure 4: The integrated effect of feldspar (FD), Filter cake (FC) treatments and their combinations with biofertilizer on soil reaction pH (A), Electrical conductivity EC (B), organic matter (OM) (C), available nitrogen (D), available phosphorus (E), and available potassium (F) (mg kg^− 1^) after successive season 2022/2023. Where MBc(-) = Without biofertilizer (*Bacillus cereus*); MBc(+) = With biofertilizer (*Bacillus cereus*); Control = Non-fertilizer; 100FD = 100% Feldspar; 100FC = 100% Filer Cake; 75FD + 25FC = 75% Feldspar + 25% Filer Cake; 25FD + 75FC = 25% Feldspar + 75% Filer Cake; 50FD + 50FC = 50% Feldspar + 50% Filer Cake. The values shown in figure are means three replicates. Means followed by the same letters are non-significantly different (*p ≤ 0.05*).

### Correlation between soil properties and plant characteristics

**Fig. 5:**
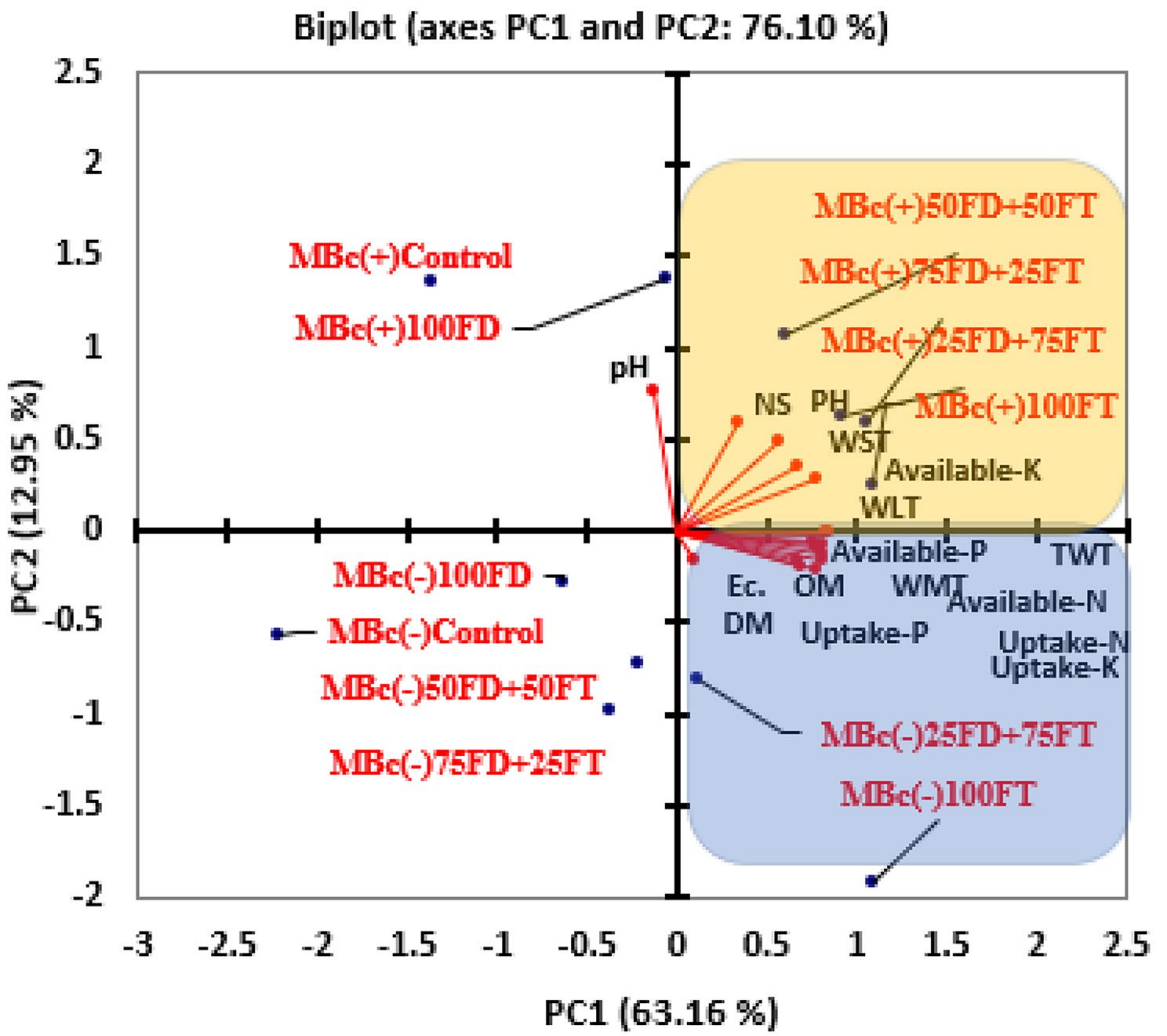
Principal component analysis (PCA) between soil properties and potato traits. Where MBc(-) = Without biofertilizer (*Bacillus cereus*); MBc(+) = With biofertilizer (*Bacillus cereus*); Control = Non-fertilizer; 100FD = 100% Feldspar; 100FC = 100% Filer Cake; 75FD + 25FC = 75% Feldspar + 25% Filer Cake; 25FD + 75FC = 25% Feldspar + 75% Filer Cake; 50FD + 50FC = 50% Feldspar + 50% Filer Cake; pH = pH value; Ec.= Soil electrical conductivity; OM = organic matter; Available-N = Available Nitrogen; Available-P = Available Phosphor; Available-K = Available Potassium; Uptake-N = Nitrogen uptake;Uptake-P = Phosphor uptake; Uptake-K = Potassium uptake; PH = Plant height; NS = No of stem; DM = Dry matter of tuber; WLT = Large Tuber weight; WMT = Medium Tuber weight; WST = Small Tuber weight; TWT = on total tuber weight

The results of the Principal Component Analysis (PCA) in Fig. 5 reveal important insights: The first two principal components (PCs) collectively explained a substantial portion (76.10%) of the variation in soil and potato traits. PC1 displayed a significant positive correlation with various soil and plant characteristics. It was positively associated with soil electrical conductivity, organic matter content, available nitrogen, available phosphorus, nitrogen uptake, phosphorus uptake, potassium uptake, medium tuber weight, and total tuber weight. This suggests that these variables tend to vary together and contribute to a common underlying factor. PC2 exhibited positive correlations with available potassium, plant height, number of stems, weight of large tubers, and weight of small tubers. It also showed a significant negative correlation with pH value. This implies that these variables are interrelated and contribute to a separate factor that is distinct from PC1. Different treatments, such as 100% Filter Cake, 25% Feldspar + 75% Filter Cake, 100% Filter Cake with Bacillus cereus, 75% Feldspar + 25% Filter Cake with Bacillus cereus, 25% Feldspar + 75% Filter Cake with *Bacillus cereus*, and 50% Feldspar + 50% Filter Cake with *Bacillus cereus*, had a positive impact on nutrient availability and plant growth indicators. These treatments contributed to the observed correlations and positively influenced the measured soil and plant characteristics. In summary, the PCA results highlight the interrelationships among various soil and potato traits, with PC1 and PC2 capturing different aspects of these associations. Additionally, the mentioned treatments positively affected nutrient availability and plant growth indicators, influencing the observed correlations.

Figure 5. Principal component analysis (PCA) between soil properties and potato traits. Where MBc(-) = Without biofertilizer (*Bacillus cereus*); MBc(+) = With biofertilizer (*Bacillus cereus*); Control = Non-fertilizer; 100FD = 100% Feldspar; 100FC = 100% Filer Cake; 75FD + 25FC = 75% Feldspar + 25% Filer Cake; 25FD + 75FC = 25% Feldspar + 75% Filer Cake; 50FD + 50FC = 50% Feldspar + 50% Filer Cake; pH = pH value; Ec.= Soil electrical conductivity; OM = organic matter; Available-N = Available Nitrogen; Available-P = Available Phosphor; Available-K = Available Potassium; Uptake-N = Nitrogen uptake;Uptake-P = Phosphor uptake; Uptake-K = Potassium uptake; PH = Plant height; NS = No of stem; DM = Dry matter of tuber; WLT = Large Tuber weight; WMT = Medium Tuber weight; WST = Small Tuber weight; TWT = on total tuber weight.

## Discussion

The soil properties, including soil pH, electrical conductivity, organic matter content, and plant growth indicators, are influenced by the separate application of potassium sources and microorganisms specifically *Bacillus cereus*, as well as their combined application.

The findings demonstrated that the addition of bio-fertilizer (MBc) had a slight decrease on soil pH, whereas when organic sources of potassium fertilization were applied without MBc, a more decrease in pH was observed. This slight decrees in soil pH could be attributed to the increased soil buffering capacity, which makes it resistant to pH changes caused by MBc addition (Ali et al. 2021a). Soil pH increased by feldspar application due to its high pH level, high alkaline mineral concentrations, functional groups associated with active soil reaction (OH and COOH) and exchangeable basic cations in feldspar amendment (Al-Sayed et al. 2022; Shabrawy and Ragab 2019). The results also indicated an increase in electrical conductivity (EC) with the application of both filter cake (FC) and MBc, either individually or in combination. This outcome aligns with the findings of El-Tayeh et al. (2019), who observed a gradual increase in EC with the addition of filter mud cake at different rates. The organic matter content of the soil, which significantly influences soil fertility, biological activity, and physical and chemical properties, exhibited a more pronounced increase with FC application, likely due to its high organic matter content compared to other sources (El-Tayeh et al. 2019). In a study conducted by El-Tayeh et al. (2019) it was found that the organic matter content increased by 6.51%, 19.44%, and 32.53% with the addition of 10%, 30%, and 50% FC, respectively, compared to the control group.

Furthermore, the addition of filter cake (FC), alone or combination with bio-fertilizer (MBc), as an organic material can influence soil nutrient availability and plant uptake. A study by Ali et al. (2021a) supports these findings and highlights the potential of FC and MBc in positively altering fertilization practices and facilitating nutrient cycling in the soil. As anticipated, the presence of active potassium solubilizing bacteria plays a crucial role in decomposing both native and added organic matter in the soil. This decomposition process leads to the release of more nutrients, making them readily available for plant uptake. A study by Bagyalakshmi et al. (2017) supports this notion and highlights the role of potassium solubilizing bacteria in improving nutrient availability for plants. Also, the inoculation of bacteria with FC can potentially enhance root growth (Al Methyeb et al. 2023), leading to increased respiration and organic acid production (Rosa et al. 2022). This, in turn, promotes overall plant growth. These findings are supported by a study conducted by Ali et al. (2021a), which reported significant increases nitrogen (N), phosphorus (P), and potassium (K) by 94.31%, 36.86%, and 71.54%, respectively, when using the filter cake treatment compared to the untreated control.

The substantial enhancement observed in the soil properties, nutrient availability, and nutrient uptake had a significant positive impact on the growth and yield of potato plants upon the application of FC and FD fertilizers, both individually and combination with bio-fertilizer. The increase in plant height and stem numbers can be attributed to the improved uptake of potassium (K) and its role in stimulating cell division, thereby promoting early growth and photosynthesis (Hasanuzzaman et al. 2018; Sardans and Peñuelas 2021). Additionally, the improvement in soil properties may have facilitated the release of more available nutrients, further supporting plant growth and development (Ali et al. 2021b; Bindraban et al. 2015). Basha (2011) conducted a study investigating the impact of filter mud cake at different rates, ranging from 5.95 to 23.8 tons per hectare, without the use of bio-fertilizers. The results of the study indicated that the application of filter mud cake at these rates had a significant positive effect on the vegetative growth of plants. In a study conducted by Sakr et al. (2014), found that the inoculation of bacteria in combination with rock phosphate and feldspar resulted in significantly higher levels of phosphorus (P) and potassium (K) in various traits of roselle (*Hibiscus sabdariffa* L.) plants. The findings from Anjanadevi et al. (2016); Ali et al. (2021a) support the positive effects of bio-fertilizer with potassium solubilizing bacteria (MBc) on potato growth. Therefore, the results are consistent with the notion that the use of potassium solubilizing bacteria can have a beneficial impact on promoting the growth of potato crops. In their study, Abdel-Salam and Shams (2012) investigated the impact of inoculating potato plants with potassium solubilizing bacteria (PSB) enhance vegetative growth compared to the untreated plants in clay soil. The dry matter content of potato tubers plays a crucial role in their processing quality, particularly in terms of texture for products such as chips and French fries (Leonel et al. 2017; Sawicka et al. 2021). A higher dry matter content is preferred as it contributes to a desired crisp texture, while tubers with lower dry matter content tend to result in lower chip yield and a soggy texture, mainly due to excessive oil absorption during and/or after frying (Kita et al. 2007). Therefore, maintaining an optimal dry matter content in potato tubers is important for achieving the desired texture and overall quality of processed potato products. The application of FC100, with or without MBc, resulted in the highest increase in total potato yield compared to other treatments. These findings provide confirmation and support for our hypotheses regarding the effectiveness of FC, either alone or in combination with FD, along with biofertilizer as a promising and viable alternative to chemical fertilizers. This highlights the potential of utilizing such management practices for sustainable agricultural production. Potassium plays a crucial role in various aspects of potato growth and development (Torabian et al. 2021). It is involved in root growth, metabolic processes, and the activation of enzymes, as highlighted by Cui and Tcherkez (2021). Additionally, potassium has a significant impact on potato tuber size and overall yield, as reported by Ali et al. (2021a). Its role in photosynthesis and the transportation of carbohydrates to tubers is vital, and it facilitates the conversion of carbohydrates into starch, protein, and vitamins, as emphasized by Hasanuzzaman et al. (2018) and Johnson et al. (2022). Therefore, proper potassium fertilization is essential for tuber bulking and the composition of tubers, as mentioned by Ewais et al. (2020) and Torabian et al. (2021). The availability and appropriate application of potassium contribute significantly to achieving optimal tuber growth and quality.

The addition of 100FC along with MBc resulted in the highest relative increase in the weight of large and medium-sized potato tubers intended for export. On the other hand, the treatment of 50FD + 50FC along with MBc showed an increase in the weight of small-sized tubers compared to other treatments. The enlargement of tuber size can be attributed to the role of potassium in facilitating cell division and the process of photosynthesis (Torabian et al. 2021). Potassium is also involved in translocation through the phloem and the production of starch within storage organs (Sardans and Peñuelas 2021). Additionally, the integrated nutrient management approach involving the application of organic fertilizers may have contributed to the overall increase in different tuber grades. The increase total tuber yield and the graded weight of tubers can be attributed to multiple factors. Firstly, the combined effect of FC and bio-fertilizer inoculation may have led to an increase in the number of tubers produced which led to increase nutrient uptake and enhance plant growth through the production of plant hormones (Johnson et al. 2022). Secondly, the presence of FC, along with its nutrient content, likely contributed to the overall improvement in tuber yield (Gonfa et al. 2018). These findings align with the results obtained by Ali et al. (2021a), highlighting the importance of potassium fertilizer regimes in achieving high tuber yields and improving tuber quality in a sustainable and cost-effective manner. The combined application of FC and biofertilizers appears to be a promising approach for optimizing potato production and achieving desirable tuber characteristics.

The results of the study demonstrate that the alternative of potassium sources and the application of biofertilizer significantly impact the yield traits of potato plants. The treatment with 100% filter cake (100FC) resulted in the highest weight of large tubers during both growing seasons. The cumulative effect of potassium sources and biofertilizer showed that the combination of 100FC with biofertilizer resulted in the maximum total tuber weight. The plants under the control group without biofertilizer had the minimum total tuber weight. Similarly, the highest dry matter of potato tubers was recorded in plants treated with 75% feldspar and 25% filter cake along with biofertilizer. On the other hand, the control group without biofertilizer had the lowest dry matter of potato tubers. These findings highlight the significant influence of potassium sources and biofertilizer on yield traits, emphasizing the importance of their proper selection and application for optimizing potato production.

## Data Availability

All data available within the article.
